# Transcriptome and Comparative Gene Expression Analysis of *Sogatella furcifera* (Horváth) in Response to Southern Rice Black-Streaked Dwarf Virus

**DOI:** 10.1371/journal.pone.0036238

**Published:** 2012-04-27

**Authors:** Yi Xu, Wenwu Zhou, Yijun Zhou, Jianxiang Wu, Xueping Zhou

**Affiliations:** 1 State Key Laboratory of Rice Biology, Institute of Biotechnology, Zhejiang University, Hangzhou, People's Republic of China; 2 Institute of Insect Sciences, Zhejiang University, Hangzhou, People's Republic of China; 3 Institute of Plant Protection, Jiangsu Academy of Agricultural Sciences, Nanjing, People's Republic of China; University of Wisconsin-Milwaukee, United States of America

## Abstract

**Background:**

The white backed planthopper (WBPH), *Sogatella furcifera* (Horváth), causes great damage to many crops by direct feeding or transmitting plant viruses. Southern rice black-streaked dwarf virus (SRBSDV), transmitted by WBPH, has become a great threat to rice production in East Asia.

**Methodology/Principal Findings:**

By de novo transcriptome assembling and massive parallel pyrosequencing, we constructed two transcriptomes of WBPH and profiled the alternation of gene expression in response to SRBSDV infection in transcriptional level. Over 25 million reads of high-quality DNA sequences and 81388 different unigenes were generated using Illumina technology from both viruliferous and non-viruliferous WBPH. WBPH has a very similar gene ontological distribution to other two closely related rice planthoppers, *Nilaparvata lugens* and *Laodelphax striatellus*. 7291 microsatellite loci were also predicted which could be useful for further evolutionary analysis. Furthermore, comparative analysis of the two transcriptomes generated from viruliferous and non-viruliferous WBPH provided a list of candidate transcripts that potentially were elicited as a response to viral infection. Pathway analyses of a subset of these transcripts indicated that SRBSDV infection may perturb primary metabolism and the ubiquitin-proteasome pathways. In addition, 5.5% (181 out of 3315) of the genes in cell cytoskeleton organization pathway showed obvious changes. Our data also demonstrated that SRBSDV infection activated the immunity regulatory systems of WBPH, such as RNA interference, autophagy and antimicrobial peptide production.

**Conclusions/Significance:**

We employed massively parallel pyrosequencing to collect ESTs from viruliferous and non-viruliferous samples of WBPH. 81388 different unigenes have been obtained. We for the first time described the direct effects of a *Reoviridae* family plant virus on global gene expression profiles of its insect vector using high-throughput sequencing. Our study will provide a road map for future investigations of the fascinating interactions between *Reoviridae* viruses and their insect vectors, and provide new strategies for crop protection.

## Introduction

Rice viral diseases are major threats to rice production and have been distributed worldwide across regions depending on rice cultivation [1]. The most prevalent rice viruses are plant-infecting reoviruses in the genera *Phytoreovirus*, *Fijivirus* and *Oryzavirus* of the family *Reoviridae*. Southern rice black streak dwarf virus (SRBSDV), commonly known as Rice black-streaked dwarf virus 2 (RBSDV 2), is a novel member of the *Fijivirus* [2]. Since its first observation in 2001 in Guangdong, China, SRBSDV has spread rapidly and now causes large yield losses throughout southern and central China, northern Vietnam, and recently has been identified in northern China [3], [4] and Japan [5]. When infected with SRBSDV, rice often develops stunted stems, dark green, twisted leaves, and white waxy swellings along veins on the abaxial surface of the leaves. When infected in seedling stage, rice shows more severe stunting and occasionally dried necrotic leaves. With infections after tillering, stunting is usually not so obvious, but disrupted head development and shrunken grains are apparent in many fields.

SRBSDV is non-enveloped and has an icosahedral capsid (T = 13) composed of an outer and inner protein shells, similar to other known fijiviruses. Virions contain ten linear genomic segments (named S1, S2, S3, S4, S5, S6, S7, S8, S9, S10) of double-stranded RNA (dsRNA), which range from approximately 4.5 to 1.4 kb in size [2], [6]. Sequence comparisons and phylogenetic analyses show that SRBSDV has a high sequence similarity with RBSDV, and that segments S1 and S10 have the highest relatedness [7]. Although the P7-1 protein of SRBSDV was recently reported to induce the formation of tubular structures in insect cells and may be a virus movement protein [8], the molecular functions of the translated proteins have not been documented.

SRBSDV is transmitted by the delphacid member white-backed planthopper (WBPH), *Sogatella furcifera* (Hemiptera: Delphacidae), in a persistent-propagative manner [2]. WBPH is one of the most economically important insect pests in Asian countries [9] and as an oligophagous plant-feeder, WBPH can cause great harm by direct feeding or vectoring SRBSDV to crops, such as rice, wheat and maize [2], [10]. WBPH is known for its long-distance migratory habits, the areas affected migration have been regarded to be the central and southeastern part of China, and Vietnam, and these areas are consistent with spread and emergence of SRBSDV during the past years [11], [12].

The ecological and physiological perspectives of WBPH and other hemipteran insect pests have been extensively studied [13], [14], but molecular mechanisms whereby the insect causes crop damage and yield losses are poorly understood. Recently, transcriptomes of the planthoppers *Nilaparvata lugens* and *Laodelphax striatellus* were reported using next-generation DNA sequencing techniques [15], [16]. Transcriptional response of whitefly to a geminivirus was also reported [17]. These papers provide considerable information relevant to the genomics of planthoppers and whiteflies, and also generate some insight into the molecular mechanisms of insect defense against virus infection. However, the genomic resources available for WBPH are still scarce and searches in GenBank identified only about 156 WBPH ESTs. Hence, this limited amount of data provides little information for transcriptional, proteomic, and gene functional analysis of WBPH and almost no information is available to dissect the complicated interactions between the newly emerged SRBSDV and its vector WBPH. However, next-generation high-throughput DNA sequencing technique has provided unprecedented fascinating opportunities for gene discovery of WBPH and detection of the global transcription responses to SRBSDV infection.

Here, we constructed two transcriptomes of WBPH and profiled the alternation of gene expression in response to SRBSDV infection at the transcriptional level. As a whole, 81388 distinct unigenes have been identified and the results indicated that SRBSDV infection can potentially perturb primary metabolism and the ubiquitin-proteasome pathway of WBPH and activate immune regulatory systems, such as RNA interfering, autophagy and antimicrobial peptide production. To our knowledge, this is the first report to define the WBPH transcriptome.

## Results and Discussion

### Illumina sequencing and reads assembly

**Figure 1:**
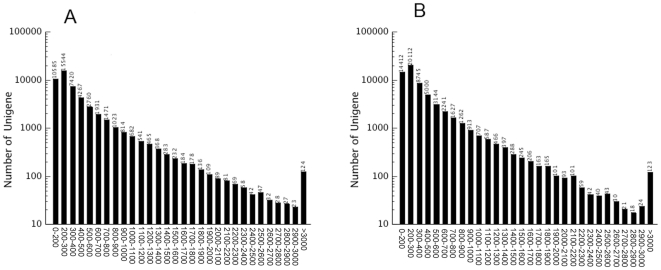
Length distribution of unigenes in the assembled transcriptomes. The x axis shows the lengths of unigenes calculated in our library and the y axis shows the number of unigenes. (A) Viruferious *Sogatella furcifera*. (B) Non-viruferious *S. furcifera*.

As described in the methods, viruliferous and non-viruliferous WBPH whole body cDNA libraries were subjected to Illumina sequencing platform, resulting in 40,665,360 and 39,135,954 reads, respectively. After cleaning and quality checks, short sequences were assembled, resulting in 136031 and 144604 contigs and data are archived at the NCBI Sequence Read Archive (SRA) under accession number SRP009194 (http://www.ncbi.nlm.nih.gov/sra). Using paired end-joining and gap-filling methods, these contigs were further assembled into 241,380 scaffolds with a mean length of 295 base pair (bp). After clustering the scaffolds with the nucleotide sequences available at NCBI, sequence data from the two libraries were combined, and 81388 unigenes were finally obtained with a mean length of 555 bp (Table 1). The length distribution of total unigenes had similar patterns between viruliferous and non-viruliferous WBPH samples, suggesting there was no bias in the construction of the cDNA libraries (Figure 1). However, some unigenes were obtained only from viruliferous or non-viruliferous samples (data not shown) and we believe these differences may be caused by distinctions that arise from long-term ecological adaptation to virus infection. All files of the assembled contigs and scaffolds from viruliferous, non-viruliferous WBPH, and the combined EST libraries are available upon request.

**Table 1:** Summary statistics for *Sogatella furcifera* genes resulting from Illumina deep sequencing.

	Viruliferous WBPH	Non-viruliferous WBPH	Combined
Contigs	144204	136031	280235
Length of all contig (nt)	31,007,772	29,013,298	60,021,070
Mean length of contigs	228	201	-
GC percentage	45.99%	44.94%	-
Scaffolds	132,907	108,473	241,380
Mean length of scaffolds	304 bp	281 bp	295 bp
All unigene	69890	61376	81388
Length of unigene	433 bp	408 bp	555 bp

### Annotation of predicted proteins

**Figure 2:**
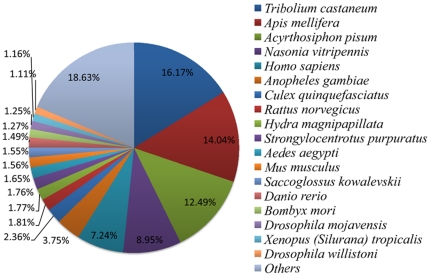
Species distribution of unigene BLASTX results against the NCBI-NR protein database with a cutoff E value <10^−5^. Different colors represent different species. Species with proportions of more than 1% are shown.

To annotate the unigenes, we searched reference sequences using BLASTX against the non-redundant (nr) NCBI protein database with a cut-off E-value of 10^−5^. A total of 28909 (35.52% of all distinct sequences) unigenes provided a BLAST result (Table S1). The species distributions of the best match result for each sequence were shown in Figure 2 and Table S2. The sequences of WBPH had a 16.17% matches with the red flour beetle (*Tribolium castaneum*) sequences, followed by 14.04% and 12.49% with honey bee (*Apis mellifera*) and the pea aphid (*Acyrthosiphon pisum*), respectively. It was surprising that WBPH shared the highest similarity with the red flour beetle in the BLAST annotation. A similar pattern has also been reported in the brown planthopper (*Nilaparvata lugens*) with the transcriptome of *N. lugens* having a similarity of 18.89% matches with the red flour beetle sequences, 14.80% with the body louse (*Pediculus humanus corporis*) and 13.19% with the pea aphids, respectively [15].

### Gene ontology (GO) classification

**Figure 3:**
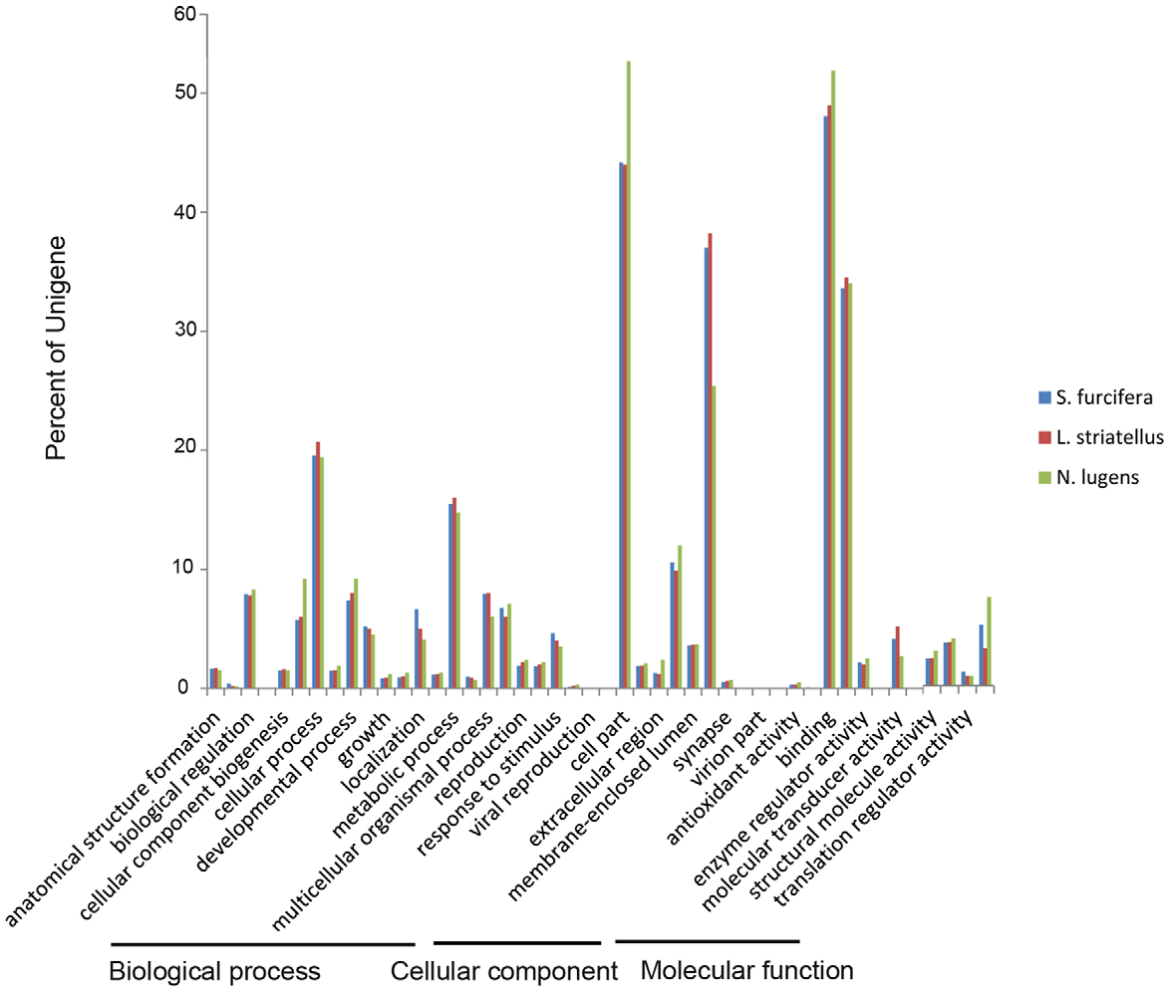
Histogram presentation of GO classification of putative functions of genes from three rice planthoppers. The functions of genes identified cover three main categories: biological process, cellular component, and molecular function. The y axis indicates the percentage of a specific category of genes in that main category.

**Figure 4:**
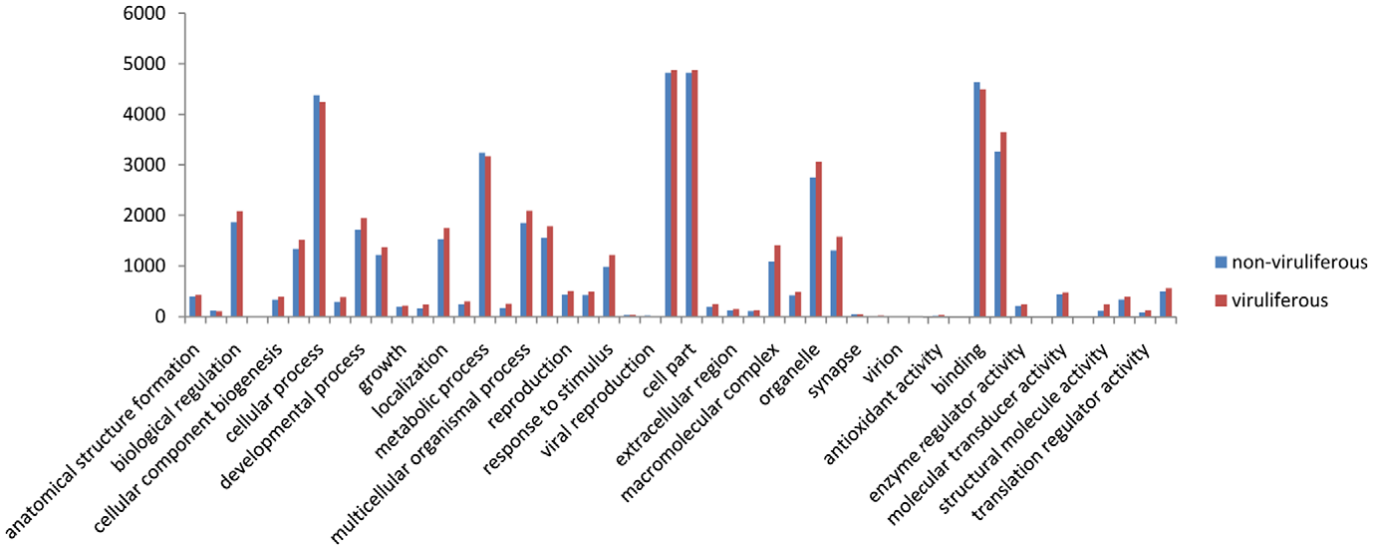
Comparison of GO classification of putative functions of genes from nonviruliferous and viruliferous *Sogatella furcifera* samples. The x axis shows subgroups of molecular functions from GO classification and the y axis shows the number of the matched unigene sequences.

We used the GO assignment to classify the functions of predicted WBPH unigenes. GO is a gene functional classification system which offers a dynamic-updated controlled vocabulary and a strictly defined concept to comprehensively describe properties of genes and their products in any organism [18]. GO has three ontologies: molecular function, cellular component and biological process. Among 30,987 annotated unigenes, approximately 18045 (58.23%) of the unigenes could be annotated in GO based on sequence homology (Table S3). When compared with the other two rice planthoppers (*N. lugens* and *L. striatellus*), WBPH had a very similar GO distribution (Figure 3). This distribution is quite reasonable given that the three sap-feeding planthopper species not only belong to the same family *Delphacidae* in the evolutionary level, but also have similar ecological niches in the rice eco-system. In addition, GO analysis also showed a similar distribution of gene functions for non-viruliferous and viruliferous WBPH (Figure 4), indicating that the number of genes expressed in each GO category was not significantly affected by SRBSDV infection.

### Putative molecular markers

**Figure 5:**
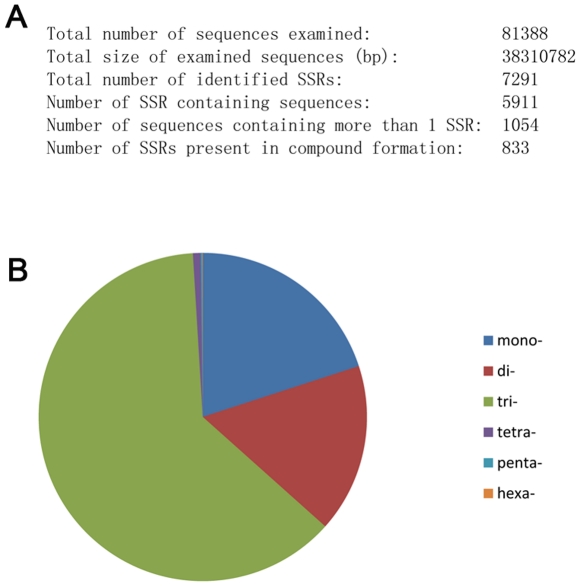
Frequency of microsatellite classes in the transcriptome of *Sogatella furcifera*. (A) Summary of SSR analysis resulting from MISA software prediction. (B) The mono- to hexanucleotide microsatellite distributions in the transcriptome sequence of *S. furcifera*.

Molecular markers are widely applied in the study of insect evolution and differentiation. As mentioned before, WBPH is well known for its yearly migration across Asian countries, resulting in the spread of SRBSDV in East Asia. A critical problem in the study of WBPH migration is lack of an effective molecular marker, which would be helpful for define of migration pathways. By using de novo assembly of transcriptome sequences, we have identified 7291 simple sequence repeats (SSRs) or microsatellites in WBPH. According to predictions, about 7.26% of protein-coding sequences possessed such repeats, of which 62.43% were trinucleotide repeats, with (AAG) _n_ being the most frequent motif (39.8%), followed by 16.62% dinucleotide and 0.7% tetranucleotide repeats (Figure 5, Table S4). The results are consistent with the recent reports indicating that trinucleotide repeats are the most abundant microsatellites in coding ESTs [19], [20]. Previous work revealed that (AAC) _n_ is the most frequent motif in *L. striatellus* [16]. The different predicted trinucleotide repeats in the two transcriptomes defined for the two available rice planthopper genomes may reflect distinct adaptive behaviors of these related insects. The large numbers of potential molecular markers found in our study will be particularly useful for future genetic mapping, parentage analysis, genotyping and gene flow studies of these species.

### Global patterns of gene expression in response to SRBSDV infection

**Figure 6:**
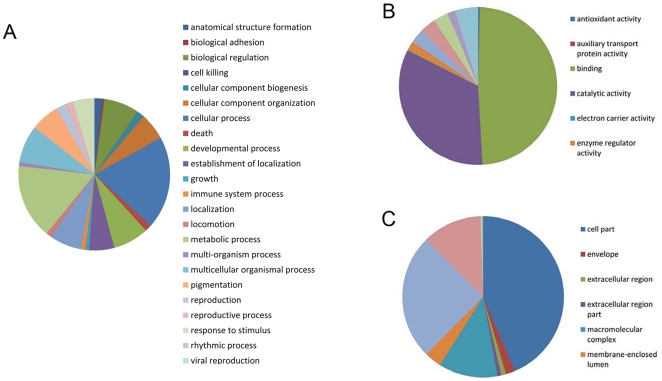
Distribution of the significantly up- and down-regulated transcripts in the subclasses of GO classification. (A) Biological process. (B) Molecular function. (C) Cellular component.

**Figure 7:**
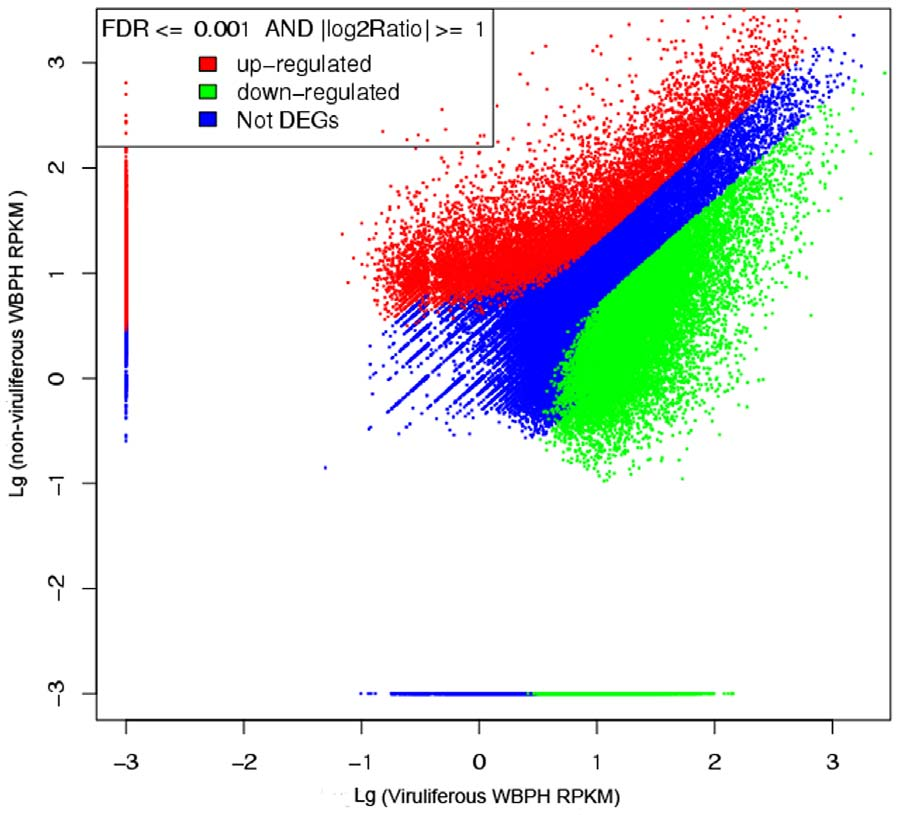
The bitmap of differentially expressed genes. The red and green points represent genes down-regulated and up-regulated genes in the viruliferous WBPH, respectively; the blue points represent genes that have no differences in regulation based on the criterion of FDR<0.001 and an absolute value of the log_2_ ratio >1.

**Figure 8:**
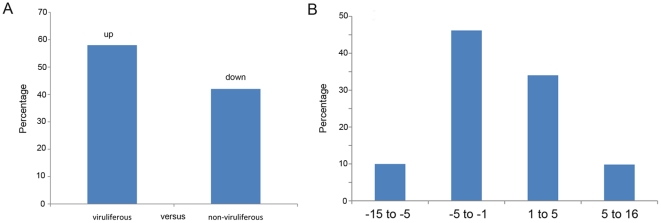
Summary of differentially expressed genes between the two libraries. (A) Summary of the percent of differentially expressed genes in the Southern rice black-streaked virus infected *Sogatella furcifera*. (B) Fold change distribution of differentially expressed genes.

To identify differentially expressed genes in response to SRBSDV infection, the numbers of clean tags for each gene were calculated, and then individual sets of reads were mapped back to the previously assembled transcript and counted as a proxy for gene expression. The differentially expressed transcripts between the two samples were identified using an algorithm developed by Audic et al [21]. The expressed transcripts were assigned into groups according to their functions, such as biological process (3315 sequences, Figure 6a, Table S5), cellular component (2965 sequences, Figure 6b, Table S5) and molecular function (3721 sequences, Figure 6c, Table S5), and the distribution of every ontology was shown in Figure 6. Totally, 58% of the differentially expressed genes were up-regulated in the viruliferous WBPH (Figure 7 and Figure 8A). The detected fold changes (log_2_ ratio) of gene expression ranged from −15 to +16, and more than 80% of the genes were up- or down-regulated between 1.0 to 5.0 fold (Figure 8B). Among the molecular function assignments, a high percentage of genes were assigned to cellular and metabolism process genes (35.6%), and 11.8% were related to biotic stimulus genes (Figure 6).

To validate the digital gene expression (DGE) result, we compared the expression profiles of the non-viruliferous and viruliferous WBPH using qRT-PCR. We selected 42 unigenes randomly, 33 of which demonstrated a concordant direction of change for both DGE and qRT-PCR (Table S6). Of the selected unigenes, nine were inconsistent between DGE and qRT-PCR. This difference might be caused by a lower sensitivity of qRT-PCR than DGE, and read coverage may be uneven across the transcript length, owing to sequencing biases [22], [23]. Nevertheless, qRT-PCR analysis confirmed the direction of change detected by DGE analysis, indicating that our results are reliable.

### Down-regulation of primary metabolism related genes

**Figure 9:**
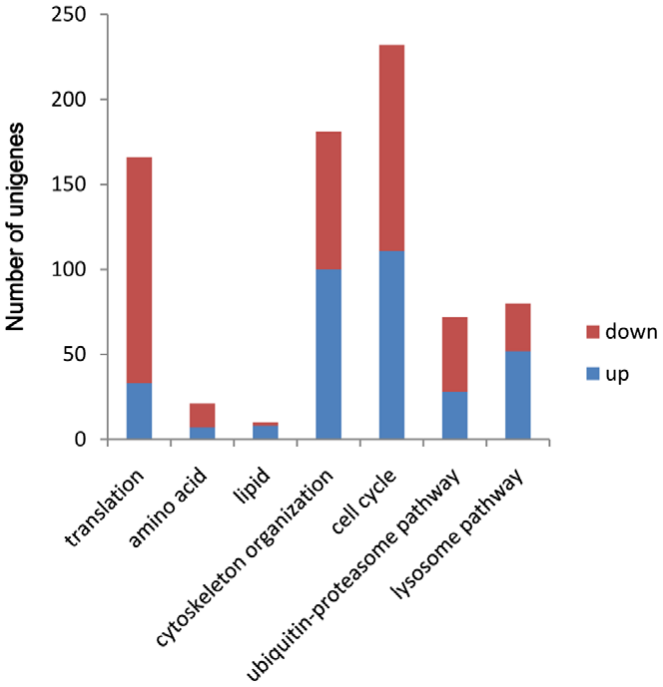
Regulated genes of viruliferous *Sogatella furcifera* involved in interesting pathways. Up-(blue) and down-regulated (red) unigenes were quantified. Regulated genes involved in primary metabolism (translation, amino acids, and lipid), cytoskeleton organization, ubiquitin-proteasome protein degradation and immune response are shown.

As mentioned above, viral infections cause dramatic changes in cellular and metabolic processes. For the primary metabolism analyses, 225 out of 3315 genes (6.8%) were down-regulated in viruliferous WBPH, most of which were involved in translation, amino acid metabolism, and biosynthesis of ribosomes, spliceosomes and aminoacyl-tRNAs (Table 2 and Figure 9). These results suggest that protein synthesis and amino acid metabolism of viruliferous WBPH were inhibited by SRBSDV infection, which are consistent with previous reports in wasps (*Campoletis sonorensis*) and whiteflies [17], [24]. In wasps, when infected by a polydnavirus, translation of specific growth-associated host proteins was inhibited [24]; and in whiteflies, the primary metabolism genes were dramatically down-regulated by Tomato yellow leaf curl China virus (TYLCCNV) infection [17]. In addition, our results indicated that a large percentage of the genes involved in lipid metabolism and lipogeneic compound metabolism were up-regulated (Table 2 and Figure 9). In contrast to whiteflies, TYLCCNV infection causes the down-regulation of lipid metabolism. A possible explanation for this phenomenon is the differences in replication styles of the two viruses. SRBSDV, a typical dsRNA virus, replicates their genomes strictly in the cytoplasm, and the lipid biosynthesis and related pathways are necessary for membrane proliferation that occurs in infected cells. TYLCCNV is a DNA virus that enters nucleus directly for its genome replication, membrane proliferation is less extensive. Effects on lipid metabolism have also been reported in other virus infections, for instance, human cytomegalovirus (CMV) infection resulted in increases in the flux of the fatty acid biosynthesis pathway genes that were essential for optimal viral growth in fibroblasts [25]. Furthermore, Hepatitis C virus has been shown to co-opt the prenylation pathway to promote the efficient replication of its genome and possibly encapsidation [26], [27]. All of these observations together with our data suggest that perturbation of lipid metabolism in a range of virally infected cells is a hallmark of cellular changes associated with viral infection.

**Table 2:** Expression profiles of genes involved in the primary metabolism pathway of viruliferous *Sogatella furcifera*.

Category or gene ID	Homologous function^a^	Species	FC^b^
**Glycolysis, protein metabolism**			
Unigene 15608	Triosephosphate isomerase B	*Danio rerio*	1.1
Unigene 78123	NADP-specificglutamate dehydrogenase	*Botryotinia fuckeliana*	−5.1
Unigene 9921	Glutathione S-transferase	*Drosophila melanogaster*	−2.45
Unigene 9674	Transaldolase	*Saccharomyces cerevisiae*	−3.9
Unigene 9668	succinate dehydrogenase	*Periplaneta Americana*	−2.19
Unigene 5478	cytochrome b small subunit	*Drosophila melanogaster*	−1.59
**Lipid metabolism**			
Unigene 65051	Fatty acid synthase	*Homo sapiens*	3.94
Unigene 34815	Acyl-CoA synthetase	*Xenopus laevis*	3.27
Unigene 13727	2,4-dehydrocholesterol reductase	*Macaca fascicularis*	1.2
Unigene 71132	hypothetical protein TcasGA2	*Tribolium castaneum*	2.71

a The functions of homologous gene.

b FC, fold changes (log_2_ ratio) in gene expression.

### Perturbance of the cell cycle and ubiquitin-proteasome pathways

Viruses depend on host's machineries to replicate and express their genomes, and replicating cells have large pools of deoxynucleotides and high levels of key enzyme activities that viruses exploit during replication [28]–[30]. So viruses have developed strategies to regulate the host cell cycle to facilitate their replication. Our results indicated that 231 out 3315 genes (7.0%) related to cell cycle were altered (Table 3). Compared with the mitotic (M) phase, there were more up-regulated genes during inter-phase according to our DGE results (data not shown). Meanwhile, pathway enrichment analyses also showed that gene expression patterns in the ubiquitin-proteasome pathway were significantly altered. The proteasome is the major non-lysosomal proteolytic machine in eukaryotes [31]. In animals and plants, perturbation of the ubiquitin-proteasome pathway has already been shown to be caused by many viruses [32], [33]. For example, Adenovirus E1A protein can directly interact with the 19 S proteasome regulatory components (S4 and S8), that are involved in regulation of the activities of 26S proteasome [34]. Inhibition of the proteasome by different chemical compounds not only impairs entry but also affects RNA synthesis and subsequent protein expression in Coronavirus infections [35]. Ubiquitination and deubiquitination of the nucleoprotein (NP) were also reported to regulate the replication of Influenza A virus RNA [36]. In the plant phyla, the geminivirus BSCTV C2 protein was reported to attenuate the degradation of SAMDC1 and suppresses DNA methylation-mediated gene silencing by inhibiting 26 S proteasome pathway in *Arabidopsis* [37]. Also in *Arabidopsis*, perturbation of ubiquitin-proteasome system affects accumulation of Turnip yellow mosaic virus (TYMV) RNA-dependent RNA polymerase during viral infection [38]. In our work, 72 out of 3315 differentially expressed genes were related to the ubiquitin-proteasome pathway, and a majority of these were down-regulated (Table 3 and Figure 9). The ubiquitin-proteasome pathway is involved in regulation of metabolic adaptation and immune responses and may function by degrading numerous short-lived proteins, such as regulatory proteins [39]–[41]. Indeed, all of these pathways were significantly affected in viruliferous WBPH.

**Table 3:** Expression profiles of genes in the ubiquitin-proteasome and cell cycle pathways.

Category or gene ID	Homologous function^a^	Species	FC^b^
**Ubiquitin-proteasome**			
Unigene 79286	26 S proteasome regulatory subunit rpn-8	*Neurospora crassa*	−4.2
Unigene 15453	26 S proteasome non-ATPase regulatory subunit 3	*Anopheles gambiae*	−1.36
Unigene 12709	26 S proteasome non-ATPase regulatory subunit 12	*Pongo abelii*	−2.77
Unigene 2468	26 S proteasome non-ATPase regulatory subunit 6	*D. melanogaster*	−2.34
Unigene 43762	26 S proteasome non-ATPase regulatory subunit 2	*Rattus norvegicus*	−2.16
Unigene 45424	26 S protease regulatory subunit 4	*D. melanogaster*	−3.55
Unigene 66815	Probable proteasome subunit beta type-2	*Neurospora crassa*	−4.9
Unigene 80945	Proteasome component PRE2	*Saccharomyces cerevisiae*	−5.9
**Cell cycle**			
Unigene 5518	S-phase kinase-associated protein 1	*Xenopus laevis*	1.15
Unigene 63682	cyclin-dependent serine/threonine-protein kinase	*Dictyostelium discoideum*	3.59
Unigene 6624	Checkpoint protein HUS1	*Mus musculus*	1.29

a and

b , see Table 2.

### Effects on the transcription of host cytoskeleton regulated genes

Among the differentially expressed genes in the viruliferous WBPH, 5.5% (181 out of 3315) genes related to cytoskeleton organization were altered, including 70 microtubule cytoskeleton organization genes and 74 actin cytoskeleton organization genes. Cytoskeleton-dependent intracellular transport is a common strategy for virus transport to intracellular destinations [42]–[47]. For example, association with microtubules is necessary for the release of Rice gall dwarf virus (RGDV) from cultured insect vector cells [48]; the Pns10 of Rice dwarf virus (RDV) induces tubular structures that facilitate virus spread in the vector *Nephotettix cincticeps* [49]. RGDV and RDV, together with SRBSDV that we studied in this work, all belong to the *Reoviridae* family, so a potential similar cytoskeleton regulatory pathway may exist among these viruses. Meanwhile, according to a recent work using a recombinant insect baculovirus expression system, the SRBSDV P7-1 protein formed tubular structures in insect cells [8]. By integrating these data with our transcriptome results, it is reasonable to believe that the dynamics of cytoskeleton has a major role in virus infection.

### Activation of cellular and humoral immune responses and RNA interfering pathway

Cellular and humoral responses are major response systems used by insects against microbial infection [50]–[53]. Among the differentially expressed genes in viruliferous WBPH, many genes related to cellular and humoral immune response were up-regulated (Table 4 and Figure 9). For example, 65% (52 out 80) of the unigenes were up-regulated in the lysosome pathway. B-cell lymphoma 6 protein (BCL-6), an important regulation factor in humoral immune responses, is believed to be involved in germinal center B-cell functions and the immune response [54], [55]. This protein acts as a sequence-specific repressor of transcription, and has been shown to modulate the transcription of START-dependent IL-4 responses of B cells and altered expression of BCL-6 leads to modulated IL-4 levels and increases in the immune response [56]. Also, among the differentially expressed genes, antimicrobial peptide and melanization-related product synthesizing genes were also up-regulated in the WBPH. Phospholipase A2 products are considered to interfere with viral infection and it has been found that several phospholipases A2 can efficiently protect host cells from HIV-1 replication [57], [58]. Down-regulation of the phospholipase A2 inhibitor subunit B (Table 3) may be helpful for the releasing of active phospholipases A2, and thus improve host defenses against virus attacks.

**Table 4:** Expression profiles of genes in the immune response pathway.

Category or gene ID	Homologous function^a^	Species	FC^b^
**RNA interfering**			
Unigene 59008	Endoribonuclease Dicer-2	*Mus musculus*	3.5
Unigene 63594	Endoribonuclease Dcr-1	*Gallus gallus*	3.8
Unigene 9996	Protein argonaute-2	*Drosophila melanogaster*	−1.61
**Immunity**			
Unigene 29497	RanBP-type and C3HC4-type zinc finger-containing protein	*Homo sapiens*	4.45
Unigene 6603	Adenosine deaminase	*Xenopus laevis*	2.348
Unigene 18472	Disintegrin and metalloproteinase domain-containing protein	*Homo sapiens*	1.76
Unigene 77216	B-cell lymphoma 6 protein homolog	*Mus musculus*	−3.1
Unigene 8447	immunoglobulin superfamily-4	*Homo sapiens*	1.8
**Autophagy**			
Unigene 6320	Autophagy protein 12-like	*D. melanogaster*	3.8
Unigene 7603	Vesicle-trafficking protein	*Danio rerio*	1.82
Unigene 64656	V-type proton ATPase subunit	*Manduca sexta*	4.34
Unigene 64690	Leucine zipper protein	*Rattus norvegicus*	6.9
**Lysosome**			
Unigene 63817	Lysosomal alpha-mannosidase	*Mus musculus*	4.45
Unigene 8125	similar to cathepsin	*Nasonia vitripennis*	2.21
**Antimicrobial peptide**			
Unigene 10741	Toll-related protein	*D. melanogaster*	1.7
Unigene 8728	Myeloid differentiation primary response protein	*Mus musculus*	2.51
**Melanization**			
Unigene 35628	Integrin alpha-4	*Mus musculus*	2.41
Unigene 63254	cAMP-dependent protein kinase catalytic subunit	*D. melanogaster*	5.9
Unigene 4776	Phenoloxidase subunit A3	*D. melanogaster*	1.29
**Antiviral**			
Unigene 64284	Phospholipase A2 inhibitor subunit	*Agkistrodon blomhoffii siniticus*	−3.06
Unigene 19480	Inhibitor of nuclear factor kappa-B kinase subunit epsilon	*Mus musculus*	3.2

a and

b , see Table 2.

RNA interference (RNAi) silences gene expression through small interfering RNAs (siRNAs) and microRNAs (miRNAs) [59], [60]. The RNAi system was initially described in plants, and in recent years, some work has revealed that RNAi is a major mechanism to target viruses in some insects [60], [61]. In *D. melanogaster*, Dicer-2 produces siRNAs, whereas Dicer-1 recognizes precursors of miRNAs. In these cases, the small RNAs are assembled with an Argonaute (AGO) protein into related effector complexes, such as the RNA-induced silencing complex (RISC), to guide specific RNA silencing [59], [61], [62]. In our transcriptome data, we found 31 records of genes (10 AGOs, 16 Dicers, 4 dsRNA binding proteins) involved in the RNA interference pathway. In the DGE results (Table 4), some involved genes were up-regulated in viruliferous WBPH, such as Dicer 1 and Dicer 2, but no substantial difference in the expression levels of putative RNAi related genes was found in the *L. striatellus* after infection with Rice stripe virus (RSV), a *tenuivirus* [16]. These differences may be caused by the diversity among the replication cycles of the two viruses or the host defense systems. The latter hypothesis is supported by recent findings that RSV and RDV (both plant reoviruses) caused different effects in the rice RNA interference pathway [63].

### Conclusion

We employed massively parallel pyrosequencing to collect ESTs from viruliferous and non-viruliferous samples of WBPH. In total, we obtained 81388 different unigenes, and have provided a major genomic resource that will be useful for subsequent investigations of the biology of WBPH and generation of new insecticide targets. We for the first time described the direct effects of a *Reoviridae* family plant virus on global gene expression profiles of its insect vector using high-throughput sequencing. The genes involved in primary metabolism, ubiquitin-proteasome, cytoskeleton dynamics and immune responses were up regulated in viruliferous WBPH. In addition, we found that an RNAi pathway exists in WBPH, and this pathway may play an important role in virus defenses. Our study will provide a road map for future investigations of the fascinating interactions between *Reoviridae* viruses and their insect vectors, and provide new strategies for crop protection.

## Materials and Methods

### Insect culture, virus, and plants

The WBPH culture used in this study originated from Jiangsu Province, China. SRBSDV-infected and un-infected rice (*Oryza sativa* cv. *Nipponbare*, K818) plants were used to feed WBPH in all experiments. For viruliferous WBPH, SRBSDV-infected rice seedlings were collected from fields and then moved to a beaker after discarding the old leaves. Then 200 WBPH second instar nymphs were swept into the beaker, and the beaker was enclosed with nylon mesh. After two days of feeding, the rice seedlings were gently turned upside-down, and the juvenile planthoppers were swept onto healthy rice seedlings. All plants were grown in soil at 25±1°C, 80% relative humidity in a growth incubator, under a long day photoperiod of 14 h in light and 10 h in darkness.

### Sample preparation and RNA isolation

After two days of feeding on SRBDV infected rice, WBPH were maintained on uninfected rice seedlings for more than 12 days (viral circulative period). PCR was then used to ensure that viral RNAs were present in the individual WBPH. During this time, non-viruliferous WBPH, as the control group, were treated identically. Approximately 100 non-viruliferous and viruliferous WBPH nymphs and adults were collected for RNA extraction. Total RNA was isolated using the TRIzol Reagent (Invitrogen, Carlsbad, CA, USA) according to the manufacturer's protocol. The concentration and quality of total RNA were determined by a Nano-Drop spectrophotometer (Thermo Fisher Scientific, Wilmington, DE, USA).

### cDNA library preparation and Illumina sequencing for transcriptome analysis

RNA was purified from 20 mg of pooled total RNA using Oligo (dT) magnetic beads and fragmented into 200–700 nucleotides length sequences in the fragmentation buffer. The cleaved poly-(A) RNA was transcribed using Oligo (dT) (Takara, Japan), and then second-strand cDNA synthesis was performed. After end repair and ligation of adaptors, the products were amplified by PCR and purified using QIAquick PCR Purification Kit to create a cDNA library. The cDNA library was sequenced on the Illumina sequencing platform (Hiseq2000). The raw reads from the images were generated by Solexa GA pipeline 1.6. After removal of low quality reads, processed reads were assembled using Short Oligonucleotide Analysis Package (SOAP) de novo software and clustered with TIGR Gene Indices (TGI) Clustering tools [64]. All raw transcriptome data have been deposited in the SRA database (NCBI). The generated unigenes larger than 350 bp were analyzed by searching the GenBank and Swissprot databases with the BLASTX algorithm. (http://www.ncbi.nlm.nih.gov/). Gene Ontology annotations and COG classfication of the unigenes were determined with the Blast2go (http://www.blast2go.org/) and Inter-ProScan software (http://www.ebi.ac.uk/Tools/pfa/iprscan/).

### Simple sequence repeats (SSR) analysis

SSRs were identified with the Microsatellite identification tool (MISA) (http://pgrc.ipk-gatersleben.de/misa/). MISA is based on Perl script designed to allow the identification and characterization of microsatellites in a comparative genomic context. Detection criteria were constrained to perfect repeat motifs of 1–6 bp and a minimum repeat number of 12, 8, 5, 5, 5 and 5, for mono-, di-, tri-, tetra-, penta- and hexa-nucleotide microsatellites, respectively.

### Analysis of differential gene expression

A rigorous algorithm was developed to identify differentially expressed genes (DEGs) between the non-viruliferous and viruliferous WBPH as described [21]. Short-read sequence data from non-viruliferous and viruliferous WBPH were mapped separately against the reference set of assembled transcripts with Burrows-Wheeler Aligner (BWA) 0.5.8a [65], relative transcript levels were output as RPKM (Reads Per Kilobase of exon model per Million mapped reads) values, which were taken into account the transcript abundance. The false discovery rate (FDR) was used to determine the threshold P-value in multiple tests. A FDR<0.001 and an absolute value of the log_2_ ratio >1 provided thresholds to determine significant differences in gene expression. The differentially expressed genes were used for GO enrichment analyses as described [18]. The correlation between two libraries was statistically assessed by calculation of the Pearson correlation coefficient (data not shown).

### Quantitative RT-PCR (qRT-PCR) analysis

To confirm the results of pyrosequencing analysis, the expression levels of 42 randomly selected genes were measured in non-viruliferous and viruliferous insects by qRT-PCR. Total RNAs from each sample were extracted using TRIzol reagent (Invitrogen) and treated with DNase I (Invitrogen) according to the manufacturer's protocol. The concentration of each RNA sample was adjusted to 1 µg/µl with nuclease-free water and total RNA was reverse transcribed in a 20 µl reaction system using the AMV RNA PCR Kit (TaKaRa). qPR-PCR was carried out on the LightCycler 480@ II with LightCycler 480@ SYBR I Master (Roche Applied Science, Basel, Switzerland) under the following conditions: 95°C for 5 min; and 40 cycles of 95°C for 10 s, 60°C for 15 s, and 72°C for 20 s, followed by melting curve generation (68°C to 95°C). Primers used in qRT-PCR for validation of differentially expressed genes were shown in Table S7. Each gene was analyzed in triplicate, after which the average threshold cycle (CT) was calculated per sample, and an endogenous 18 S rRNA gene of WBPH was used for normalization. A no template control sample (nuclease free water) was included in the experiment to detect contamination and determine the degree of dimer formation (data not shown).

## Supporting Information

Table S1 **Annotation of GenBank database searches.** This table shows the results about searching the reference sequences using BLASTX against the non-redundant (nr) NCBI database with a cut-off E-value of 10^−5^.
(XLSX)

Table S2 **Top hits obtained by BLASTX searches in the NCBI database for species distribution.**
(XLSX)

Table S3 **Combined GO analysis of transcriptome provided by Blast2Go and InterProScan.**
(XLS)

Table S4 **SSR analysis of** ***Sogatella furcifera*** **transcriptome.** General features of predicted microsatellites include the distribution to different repeat type classes, frequency of identified SSR motif and frequency of classified repeat types.
(XLS)

Table S5 **Differently expressed transcripts assignments between viruferious and non-viruferious samples according to molecular, cellular, and biological functions.**
(XLS)

Table S6 **Verification of differentially expressed genes by qRT-PCR.**
(XLS)

Table S7 **Primers used in qRT-PCR for validation of differentially expressed genes.**
(XLS)
